# Side-to-Side versus Traditional End-to-Side Approach to STA-MCA Bypass in Moyamoya Disease: A Comparative Meta-analysis

**DOI:** 10.1055/a-2945-8335

**Published:** 2026-09-14

**Authors:** David Cho, Gerardo Duran, Cory Greer, Abdurrahman F. Kharbat, Michael J. Feldman, Andrew M. Bauer, Christopher Salvatore Graffeo

**Affiliations:** 1 Department of Neurosurgery 6186 The University of Oklahoma Health Sciences CenterOklahoma CityOklahomaUnited States

**Keywords:** moyamoya disease, cerebral revascularization, anastomosis, surgical, cerebral hyperperfusion, stroke

## Abstract

**Background** Superficial temporal artery to middle cerebral artery (STA–MCA) bypass is a standard-of-care treatment for adult patients with moyamoya disease (MMD). Although the classical technique is end-to-side (E-S) bypass, the side-to-side (S-S) construct has emerged as an alternative strategy with potentially reduced risk of postoperative cerebral hyperperfusion syndrome (CHS).

**Methods** A PRISMA-compliant systematic review of MEDLINE and Embase databases was performed from inception to May 2026 identifying 72 studies. After abstract screening, 14 studies underwent full-text review, and five head-to-head studies met inclusion criteria. A meta-analysis and supplemental sensitivity analyses were conducted to assess robustness, excluding a high-variance study with disparate CHS diagnostic thresholds.

**Results** Five studies were included capturing head-to-head outcomes for a total of 495 patients (194 E-S; 301 S-S). Pooled analysis showed no significant difference in CHS incidence with S-S bypass compared with E-S bypass. Sensitivity analysis excluding a study with high CHS incidence revealed a significant reduction of CHS with S-S bypass (OR = 1.63, 95% CI: 1.19–2.25, *p* = 0.016), possibly due to decreased heterogeneity, or their inclusion of high-sensitivity diagnostic tools including laser speckle contrast imaging (LSCI) and single-photon emission computed tomography (SPECT), which may have identified a higher incidence of subclinical CHS. No significant difference was observed in the reported incidence of stroke in either model. Findings were unchanged when the analysis was limited to the non-overlapping cohorts.

**Conclusion** S-S constructs for STA–MCA in adult MMD may reduce CHS risk, but without an associated difference in risk of postoperative stroke. Future studies with standardized reporting of CHS are required to validate S-S bypass as a preferred strategy.

## Introduction

Moyamoya disease (MMD) is a rare progressive cerebrovascular disorder characterized by stenosis or occlusion of the intracranial carotid arteries and their branches, yielding a significant risk of stroke. 1 The term “moyamoya,” meaning “hazy” in Japanese, was first described in 1969 2 and refers to the characteristic “puff-of-smoke” angiographic appearance resulting from collateral vessel formation. These fragile vessels, while compensatory, are prone to rupture or inadequate perfusion, contributing to ischemic and hemorrhagic events. The cornerstone treatment for MMD in adult patients is flow augmentation via extracranial–intracranial bypass, typically via superficial temporal artery to middle cerebral artery (STA–MCA) bypass 3 4 5 6 though indirect bypass is a viable option. 7

Recently, the side-to-side (S-S) STA–MCA bypass technique has emerged as a promising approach in the management of MMD, 8 9 10 11 12 13 offering unique advantages over the traditional end-to-side (E-S) STA–MCA bypass. In contrast to the E-S approach, which involves suturing the donor STA directly to the cortical branches of the MCA while ligating the distal STA, the S-S bypass preserves integrity through the distal STA by creating an anastomosis between the peripheral walls of the proximal STA donor and the MCA cortical branch recipient.

This preservation of distal flow has been proposed to enable self-regulating blood flow adjustments, 9 10 11 12 13 reducing the incidence and severity of cerebral hyperperfusion syndrome (CHS), a serious event characterized by neurological symptoms due to an increase in cerebral blood flow (CBF) perfusion following revascularization procedures. 14 15 Additionally, the intact distal STA maintains compensatory intracranial and extracranial collateral pathways, which may further bolster cerebral perfusion and reduce ischemic risk. 9 10 11 12 13

Despite its potential benefits, the theoretical risk of decreased immediate postoperative CBF following S-S bypass compared with E-S bypass raises concerns. 10 By maintaining distal flow in the STA, this technique also introduces the possibility of competitive flow dynamics, potentially leading to insufficient blood supply to ischemic regions during the critical early postoperative period. 9 12 Such a scenario could paradoxically elevate the risk of ischemic complications, including stroke, in the early recovery phase. Correspondingly, the goal of the present meta-analysis was to compare the incidence of CHS and stroke in the postoperative setting after either E-S and S-S variants of STA–MCA bypass in adult patients with MMD.

## Methods

### Search Strategy

**Fig. 1:**
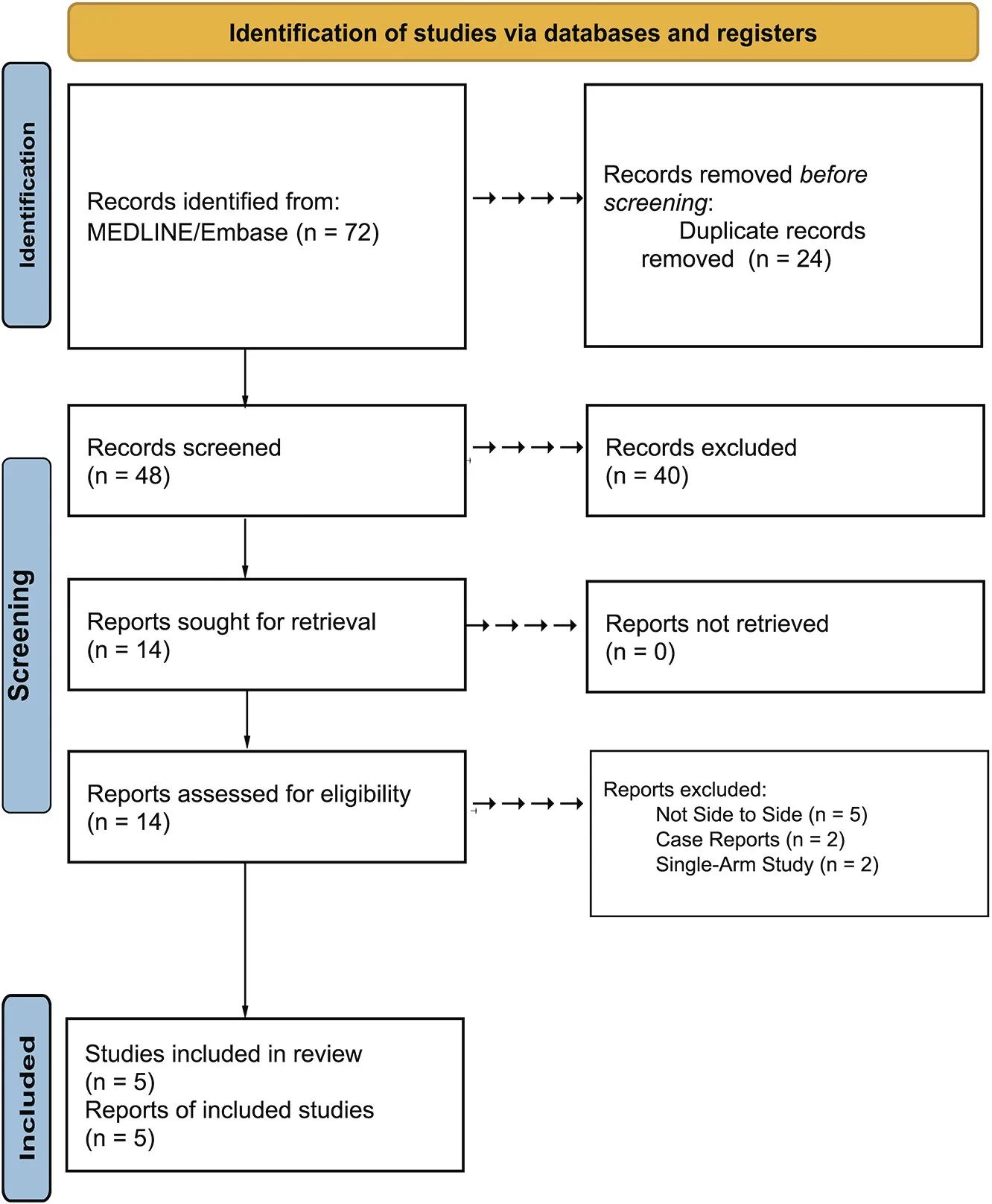
PRISMA diagram of systematic review.

A PRISMA-compliant systematic review was conducted of MEDLINE and Embase from inception through May 2026 (see Fig. 1 ). Search terms included “1d2r,” “one donor two recipient,” “one-donor two-recipient,” “one donor-two recipient,” “one-donor-two-recipient,” “1 donor 2 recipient,” “1-donor 2-recipient,” “1 donor-2 recipient,” “1-donor-2-recipient,” “side to side,” “side-to-side,” “side-side,” “s-s,” “three vessel,” “three-vessel,” “3 vessel,” “3-vessel,” “fourth generation,” “fourth-generation,” “4th generation,” “4th-generation,” “4a,” “moyamoya,” and “MMD.” Peer-reviewed studies in English met inclusion criteria if clinical outcomes of E-S and S-S STA–MCA bypass were compared in adult patients with MMD, specifically postoperative stroke incidence. The search produced 72 citations, of which 14 were selected for full-text review after screening by abstract. Ultimately, five studies met inclusion criteria for data collection, which comprised of patient age, follow-up durations, as well as incidence of CHS and postoperative stroke following E-S and S-S bypass.

### Statistical Analysis

**Fig. 2:**
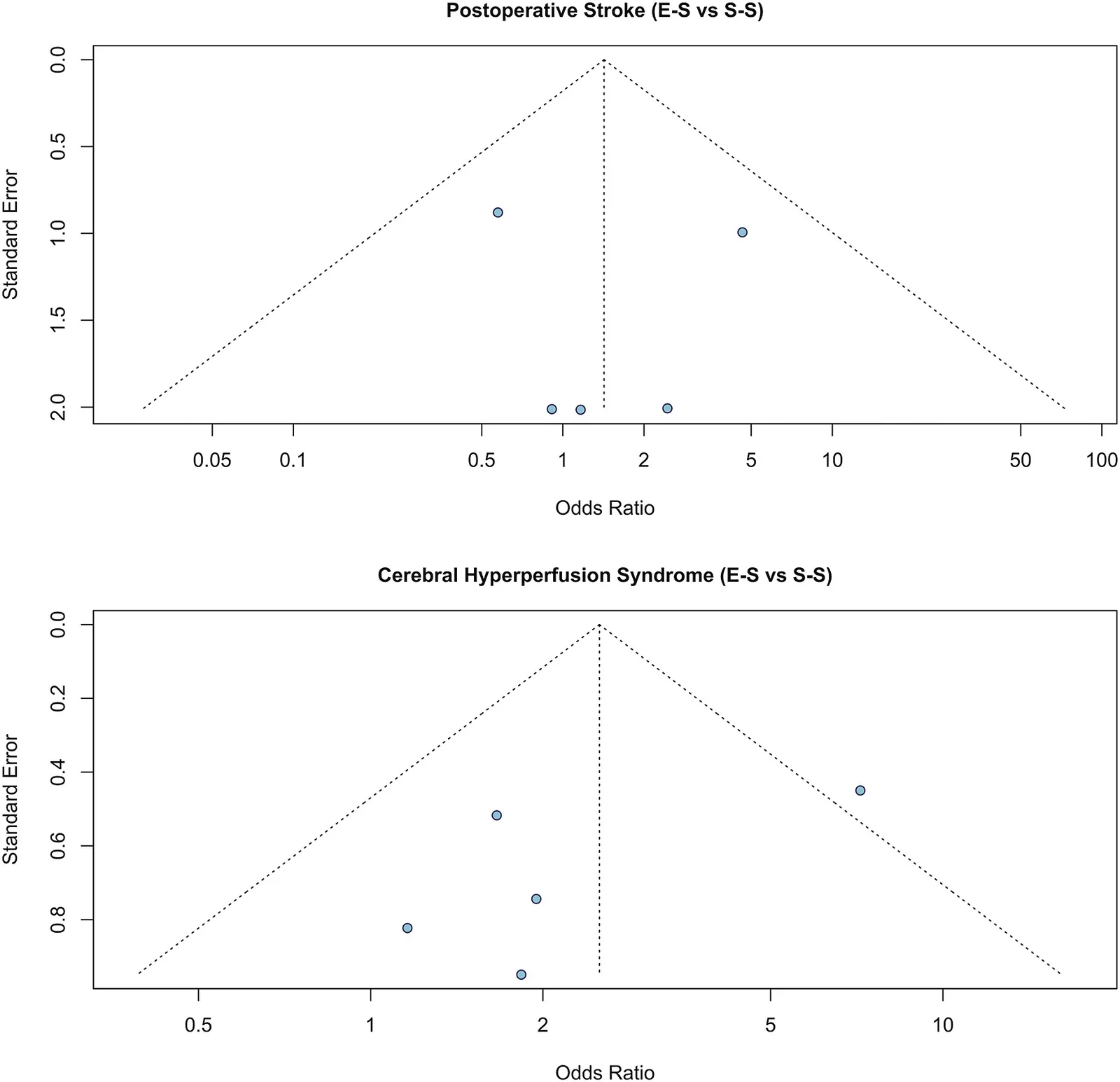
Funnel plot of included studies.

A meta-analysis was conducted using R (version 4.3.3, “Angel Food Cake”) to compare incidence of postoperative stroke between E-S and S-S configurations STA–MCA bypass adult patients with MMD. The *meta* package was employed in the calculation of pooled odds ratios (OR) and 95% confidence intervals (CI) based on the DerSimonian-Laird method for random-effects modeling. The Haldane-Anscombe correction was incorporated in the calculation of log ORs and their standard errors to account for small sample sizes and minimize bias. Sensitivity analyses were performed excluding a study that had follow-up duration less than 6 months 9 and a study with high CHS incidence likely due to utilizing LSCI and SPECT to detect CHS. 10 Because several included studies originated from overlapping investigative groups, an additional sensitivity analysis was performed to restrict cohorts to an analysis that did not overlap in terms of recruitment. Among the four cohorts reported by the Zhongnan Hospital (Wuhan University) group, the enrollment windows of Zhang et al 9 (June–December 2021), Yu et al 13 (October 2021–July 2022), and Tao et al 10 (September 2021–September 2022) overlapped, whereas that of Wan et al 12 (September 2022–May 2024) did not; the cohort of Shi et al 11 (Nanjing Drum Tower Hospital) was institutionally independent and shared no patients with any other study. Accordingly, this analysis retained three studies 10 11 12 with non-overlapping recruitment while excluding two ( Fig. 2 ).

Publication bias was assessed visually via funnel plot. Heterogeneity was assessed using the *I* ^2^ statistic, with values exceeding 50% considered markedly heterogeneous.

## Results

### Study Characteristics

Five studies met the criteria and were included, representing a total cohort of 495 patients—194 undergoing E-S and 301 patients undergoing S-S variants of STA–MCA bypass. Mean follow-up time across studies was 7 months (range, 3–12); mean patient age was 48.5 years ( Table 1 ).

**Table 1:** Study characteristics and event rates for side-to-side versus end-to-side superficial temporal artery to middle cerebral artery bypass in adult moyamoya disease.

Study	Mean F/U (mo)	Mean age (yr)	End-to-side (E-S) STA–MCA			Side-to-side (S-S) STA–MCA		
			Total ( *N* )	Postoperative stroke, *n* (%)	CHS, *n* (%)	Total ( *N* )	Postoperative stroke, *n* (%)	CHS, *n* (%)
Shi et al 11	6	48.5	27	2 (7.4%)	4 (14.8%)	23	3 (13.0%)	3 (13.0%)
Tao et al 10	12	50.38	45	3 (6.7%)	22 (48.9%)	85	1 (1.2%)	10 (11.8%)
Wan et al 12	7	47.5	48	0 (0.0%)	7 (14.6%)	118	0 (0.0%)	11 (9.3%)
Yu et al 13	7	46.7	44	0 (0.0%)	6 (13.6%)	40	0 (0.0%)	3 (7.5%)
Zhang et al 9	3	49.5	30	0 (0.0%)	3 (10.0%)	35	0 (0.0%)	2 (5.7%)
**Total/Mean**	**7.0 (3–12)**	**48.52**	**194**	**5 (2.6%)**	**42 (21.6%)**	**301**	**4 (1.3%)**	**29 (9.6%)**

Abbreviations: CHS, cerebral hyperperfusion syndrome; E-S, end-to-side; F/U, follow-up; MMD, moyamoya disease; STA–MCA, superficial temporal artery to middle cerebral artery; S-S, side-to-side.

Note: Follow-up summarized as mean (range). Event rates are expressed as *n* (%).

### Cerebral Hyperperfusion

**Fig. 3:**
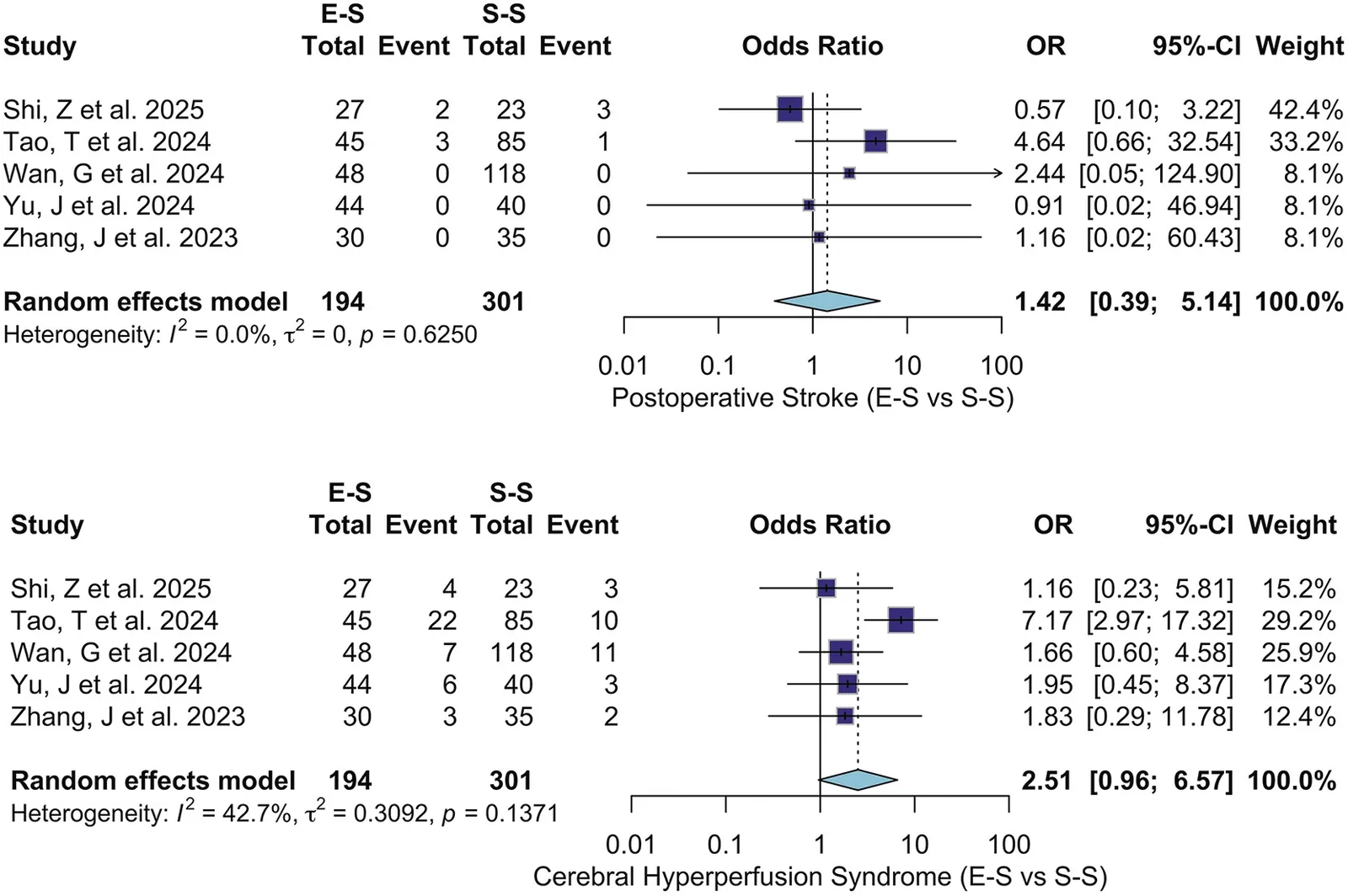
Forest plots depicting cerebral hyperperfusion syndrome (CHS) and postoperative stroke outcomes between end-to-side (E-S) and side-to-side (S-S) superficial temporal artery to middle cerebral artery (STA–MCA) bypass.

**Fig. 4:**
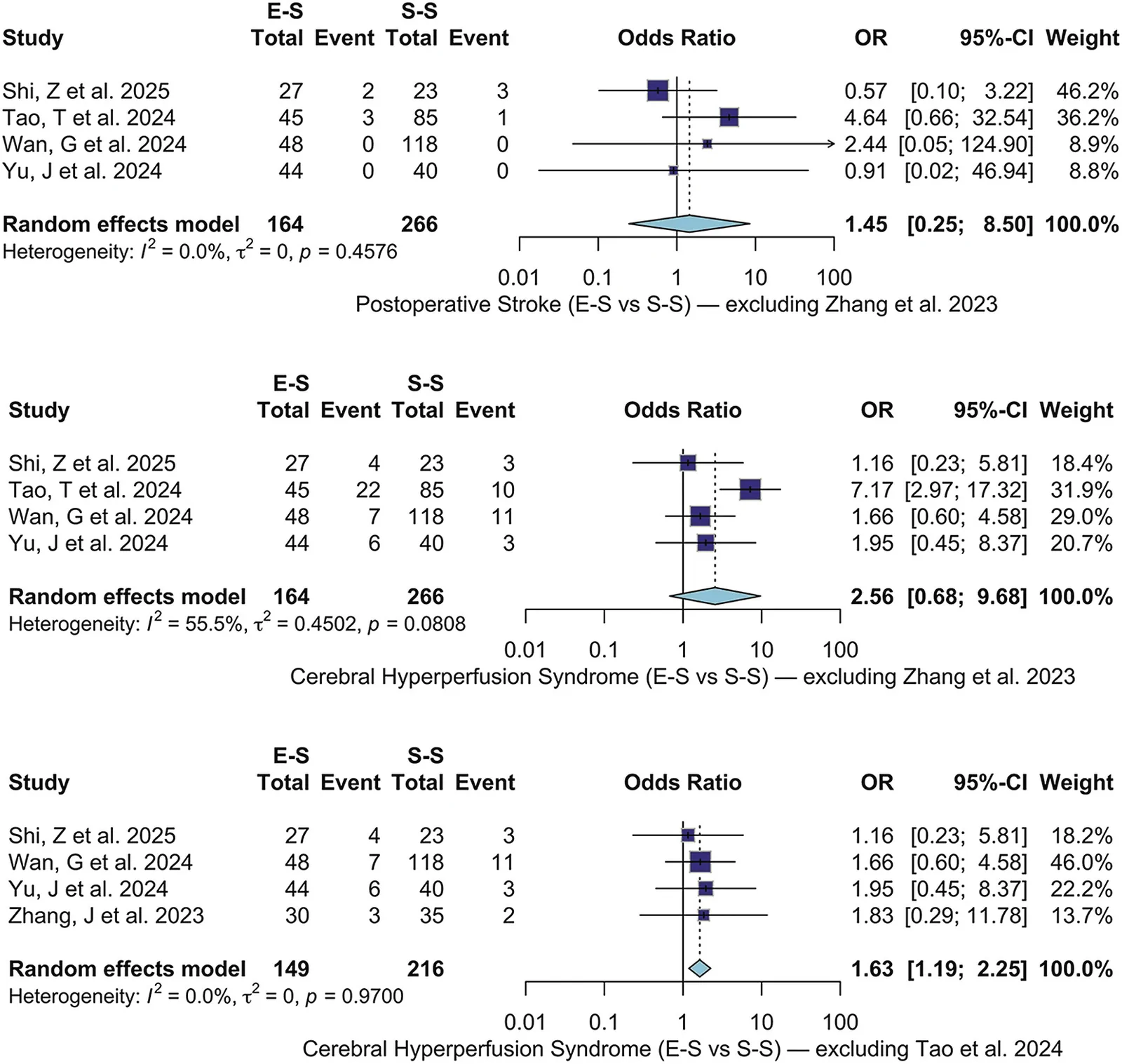
Forest plots depicting cerebral hyperperfusion syndrome (CHS) and postoperative stroke outcomes between end-to-side (E-S) and side-to-side (S-S) superficial temporal artery to middle cerebral artery (STA–MCA) bypass excluding a study that had follow-up duration less than 6 months and a study that utilized laser speckle contrast imaging (LSCI) and single-photon emission computed tomography (SPECT) to detect CHS.

**Fig. 5:**
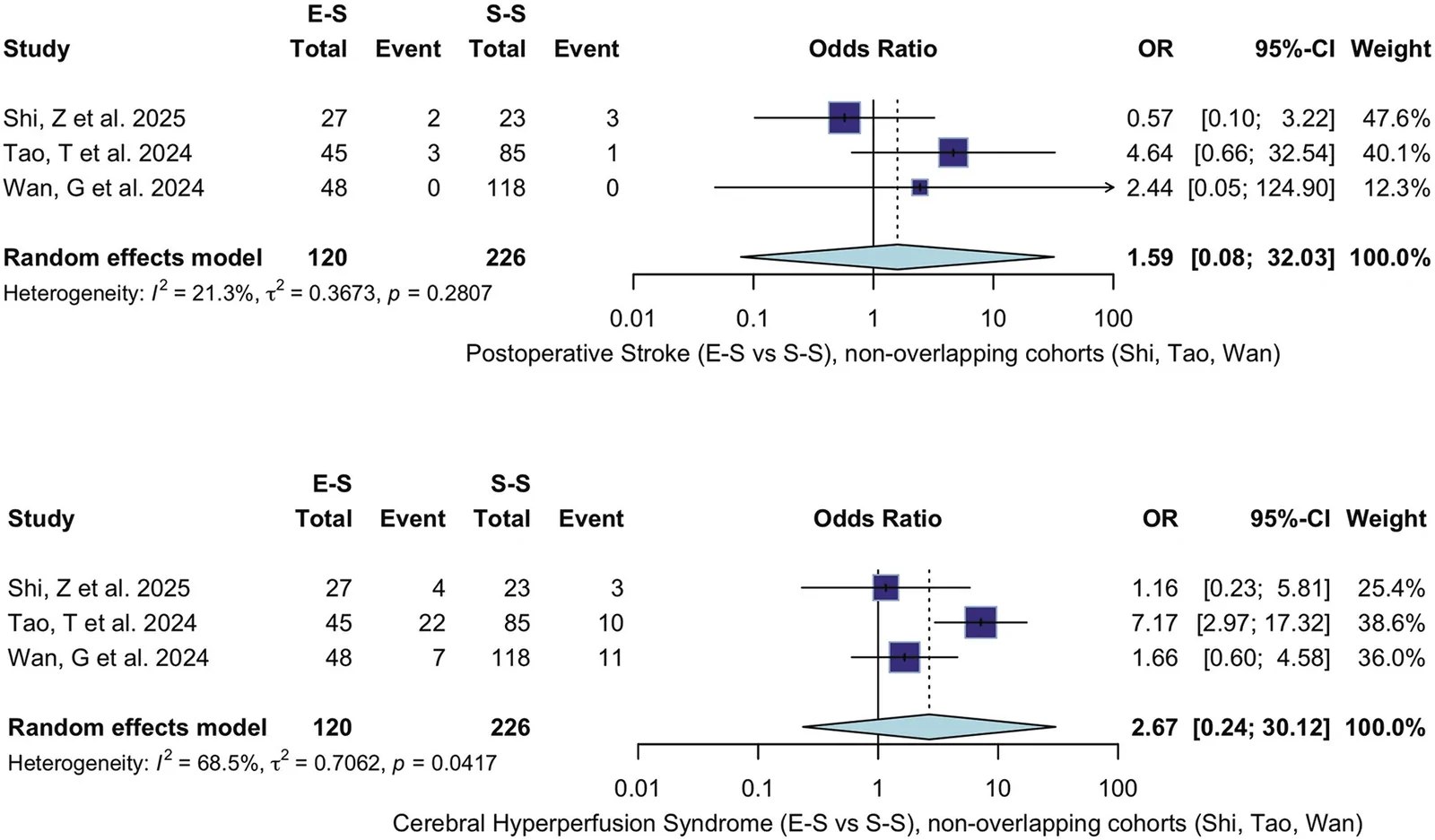
Forest plots depicting cerebral hyperperfusion syndrome (CHS) and postoperative stroke outcomes between end-to-side (E-S) and side-to-side (S-S) superficial temporal artery to middle cerebral artery (STA–MCA) bypass, restricted to the cohorts with non-overlapping patient recruitment.

Incidence rates of CHS after either E-S or S-S variant STA–MCA bypass in adult MMD patients were not significantly different ( Fig. 3 ). Exclusion of a study with follow-up duration less than 6 months 9 did not alter this finding ( Fig. 4 ). However, a sensitivity analysis excluding a study with abnormally high CHS incidence thought to be attributable to utilization of high-sensitivity diagnostic tools such as LSCI and SPECT 10 identified a significantly lower incidence of CHS after S-S bypass, as compared with E-S (OR = 1.63, 95% CI: 1.19–2.25, *p* = 0.016; Fig. 4 ). When restricted to the three non-overlapping cohorts, the difference in CHS incidence between E-S and S-S bypass remained non-significant ( Fig. 5 ), consistent with the primary analysis and indicating that the pooled estimate was not driven by patient duplication across the related cohorts.

### Postoperative Stroke

Incidence rates of postoperative stroke were not significantly different after E-S or S-S bypass ( Fig. 3 ). Exclusion of a study with follow-up duration less than 6 months 9 did not alter this finding ( Fig. 4 ). This finding was likewise unchanged in the non-overlapping-cohort analysis ( Fig. 5 ).

### Heterogeneity and Publication Bias

Heterogeneity was quantified separately for each analysis. It was negligible for the primary postoperative stroke analysis ( *I* ^2^ = 0.0%, *τ* ^2^ = 0, *p* = 0.6250) but moderate for the primary CHS analysis ( *I* ^2^ = 42.7%, *τ* ^2^ = 0.309, *p* = 0.1371). Among the CHS sensitivity analyses, heterogeneity was negligible after excluding the LSCI/SPECT study ( *I* ^2^ = 0.0%, *τ* ^2^ = 0, *p* = 0.9700), higher after excluding the short follow-up study ( *I* ^2^ = 55.5%, *τ* ^2^ = 0.450, *p* = 0.0808), and substantial in the non-overlapping cohort analysis ( *I* ^2^ = 68.5%, *τ* ^2^ = 0.706, *p* = 0.0417). However, the observed consistency across study findings is more likely attributable to the small number of included studies and limited sample sizes. Visual inspection of the funnel plot showed symmetry, suggesting minimal publication bias ( Fig. 2 ).

## Discussion

We report a novel systematic review and meta-analysis investigating differential incidence rates of CHS and stroke after S-S and E-S variants of STA–MCA bypass for adult MMD. Overall, both constructs appear to demonstrate comparable safety and efficacy profiles, with potential differential outcomes identified only through sensitivity analyses that may reflect the influence of heterogeneous diagnostic instruments, rather than a true difference between the techniques and their relative outcomes.

### S-S Bypass Mitigates CHS Risk

The pooled analysis revealed that the S-S STA–MCA bypass did not significantly reduce the incidence of CHS compared with the E-S bypass. The preserved collateral flow provided by the S-S technique is hypothesized to prevent the abrupt hemodynamic changes, 9 10 11 12 13 such as elevations in relative cerebral blood flow (rCBF) observed following traditional E-S STA–MCA bypass in MMD. 16 17 However, these findings must be interpreted cautiously due to variability in reporting standards and diagnostic thresholds for CHS across included studies.

Notably, one included study 10 used high-sensitivity diagnostic tools, such as LSCI and SPECT, which likely identified subclinical hyperperfusion cases not captured by studies relying solely on clinical symptoms or conventional imaging parameters. This increased sensitivity may account for the higher CHS rates reported (E-S: 22/45 [48.89%]; S-S: 10/85 [11.76%]). 10 Exclusion of this study was felt to describe a more clinical definition of CHS, and therefore a more appropriate comparison. Interestingly, this exclusion resulted in a significant reduction in CHS incidence following S-S bypass, as compared with E-S, at a narrower confidence interval. Taken together, these findings ballast a guarded recommendation that S-S bypass may confer a reduced risk of CHS, which may only be apparent clinically under high-risk or high-sensitivity conditions. Notwithstanding, these findings also highlight the lack of standardized definitions or diagnostic protocols for CHS, which result in challenging comparisons between studies on this core outcome.

According to a preceding meta-analysis, 18 CHS occurs in 19.9% of adults undergoing any surgery for MMD, with no significant difference detected in incidence between direct and indirect bypass procedures. The present study identified a total CHS incidence rate of 21.7% (42/194) after E-S STA–MCA bypass. Interestingly, when the high-sensitivity study 10 is excluded, the pooled CHS incidence rate is 13.4%, which is less consistent with the inferred rate for adults with MMD and more consistent with what has been reported after pooled studies of adult and pediatric patients undergoing either E-S or combined direct–indirect bypass procedures at approximately 15%. Although based on limited data and qualified interpretations, these findings present promising initial impressions that the CHS rates identified in the current sensitivity analysis may represent a more accurate picture of the true rate than much of the preceding literature.

### Potential Limitation of S-S Bypass

The defining feature of the S-S bypass is its preservation of distal donor STA outflow and resulting self-regulatory mechanism, allowing blood flow to adapt to cerebral hemodynamic demands. Greater flow through the distal STA seems to mitigate CHS incidence more dramatically than situations where less distal flow through the STA is observed. 11 Further, a preserved distal STA enables flow escape from the intracranial circulation in cases where the STA has a large diameter or decreased pressure. However, evidence of such a “distal STA steal” phenomenon has not yet been convincingly demonstrated with imaging findings in a scientific study. 9 10 Careful selection of patients, particularly those with symptomatic ischemia and a favorable donor–recipient vessel ratio may prevent the emergence of this phenomenon in the postoperative period. 9

This potential complication raises concerns regarding the increased ischemic vulnerability of the brain during the acute phase shortly after revascularization. Numerous preceding studies 9 11 have observed smaller immediate increase in CBF following S-S bypass than after E-S bypass—a difference that was not significant. 12 Longer-term cerebral cortical perfusion measured by SPECT has been reported as comparable after either S-S or E-S bypass, 10 reflecting effective redistribution of blood flow over time.

Our results suggest that the smaller immediate increase in flow following S-S bypass may be clinically negligible, given a comparable perioperative and delayed postoperative stroke risk to E-S bypass at mean 7 (3–12) months. Notably, all studies reported zero events in the E-S group except two. 10 11 Future investigation is warranted here given the low predicted event rate, and with particular attention to follow-up time after the initial 6–12 months, ideally examining late results after 2 years and adjusted for differences in clinical presentation that may influence long-term stroke risk.

### The Ideal Candidate for S-S Bypass

Although our findings only offer a speculative advantage of S-S STA–MCA bypass in reducing CHS incidence without increase in risk of postoperative stroke, there is a possibility of its latent advantages in specific clinical scenarios. The decision to pursue an E-S STA–MCA bypass or S-S STA–MCA bypass might depend on a myriad of factors but notably patients with existing scalp collateral networks off the STA branches may benefit most from the S-S technique due to its preservation of collaterals stemming from the donor branch. 8 9 Patients with poor collateral support may likewise benefit from the development of abundant scalp collaterals from the non-donor STA branch and occipital branches seen following S-S bypass. 9 Conversely, those presenting with profound ischemia yet sufficient collateral support may be better candidates for E-S bypass, where the immediate and substantial flow augmentation may provide greater benefit. The practical value of this study’s findings lies in framing the choice of bypass technique in accordance with the clinical context: S-S anastomosis in patients with favorable or recruitable collateral beds in whom CHS avoidance is preferred, and E-S anastomosis in those with severe ischemia despite adequate collaterals.

## Limitations and Future Directions

There are several limitations in this study that should be considered. First, the small number of included studies and their retrospective designs inherently limit the statistical power and generalizability of our meta-analysis. Further, while sensitivity analysis was conducted to isolate non-overlapping cohorts, unfortunately, the power of this analysis was weakened possibly contributing to false negative results. Taken together, the findings provide important insights into the comparative outcomes of S-S and E-S STA–MCA bypass techniques; however, prospective, multicenter trials with larger patient cohorts are necessary to validate these results and facilitate more robust comparisons.

Another critical limitation lies in the variability of patient selection criteria across institutions. For S-S bypass, selection of symptomatic and hypoperfused patients and donor arteries larger than the recipient arteries are critical steps in preventing potential distal STA steal. 9 A similar degree of consideration may not be given when selecting a candidate for traditional E-S bypass possibly due to familiarity, introducing bias in the comparative outcomes of the two techniques. Furthermore, the novelty of the S-S bypass contributes to variability in specific surgical technique and impacts its consistent application across different institutions. Also, adjunctive indirect bypasses may be underreported, which further constrains the ability to account for confounding factors.

Also, the variability in diagnostic and reporting standards for CHS further complicates the interpretation of this study. As discussed, one study 10 utilized high-sensitivity diagnostic tools like LSCI and SPECT, which likely identified subclinical cases of CHS not captured by studies using traditional clinical thresholds. This highlights the critical need for standardized diagnostic criteria to allow meaningful comparisons and ensure consistency in the reporting of CHS incidence.

Furthermore, this study does not include comparisons with other recent variations of direct bypass techniques, such as double-barrel bypass 7 19 20 21 22 23 24 or one-donor two-recipient anastomoses. 25 26 Future research should include these innovations to comprehensively evaluate their roles relative to traditional E-S and S-S STA–MCA bypass techniques, ultimately guiding the refinement of surgical strategies and improving outcomes for patients with MMD.

## Conclusion

We report a novel meta-analysis highlighting the potential advantages of the S-S variant of STA–MCA bypass in reducing the incidence of CHS in adult MMD patients, as compared with the traditional E-S variant. A sensitivity analysis excluding a high-variance study demonstrated lower CHS incidence with S-S bypass compared with E-S bypass; overall results were not significant, and postoperative stroke was also not significant, highlighting the need for more nuanced study with large-scale consistent outcomes in the future, as well as the fundamental safety and efficacy of both techniques. Ideally, future assessments based on multicenter, standardized, prospective studies with clear and well-validated definitions of CHS will provide more meaningful insights, with the ultimate goal of optimizing STA–MCA techniques and results on behalf of adult MMD patients.

## Declaration of GenAI Use

Generative AI (Claude, Anthropic) was used to assist with phrasing and manuscript organization. All scientific content, data interpretation, and conclusions are the authors' own, and the authors take full responsibility for the final manuscript.
